# Cerebrospinal fluid tracer efflux to parasagittal dura in humans

**DOI:** 10.1038/s41467-019-14195-x

**Published:** 2020-01-17

**Authors:** Geir Ringstad, Per Kristian Eide

**Affiliations:** 1 Department of Radiology and Nuclear Medicine, Oslo University Hospital - Rikshospitalet, Oslo, Norway; 2 Department of Neurosurgery, Oslo University Hospital-Rikshospitalet, Oslo, Norway; 3 Institute of Clinical Medicine, Faculty of Medicine, University of Oslo, Oslo, Norway

**Keywords:** Neuroscience, Neurological disorders

## Abstract

The mechanisms behind molecular transport from cerebrospinal fluid to dural lymphatic vessels remain unknown. This study utilized magnetic resonance imaging along with cerebrospinal fluid tracer to visualize clearance pathways to human dural lymphatics in vivo. In 18 subjects with suspicion of various types of cerebrospinal fluid disorders, 3D T2-Fluid Attenuated Inversion Recovery, T1-black-blood, and T1 gradient echo acquisitions were obtained prior to intrathecal administration of the contrast agent gadobutrol (0.5 ml, 1 mmol/ml), serving as a cerebrospinal fluid tracer. Propagation of tracer was followed with T1 sequences at 3, 6, 24 and 48 h after the injection. The tracer escaped from cerebrospinal fluid into parasagittal dura along the superior sagittal sinus at areas nearby entry of cortical cerebral veins. The findings demonstrate that trans-arachnoid molecular passage does occur and suggest that parasagittal dura may serve as a bridging link between human brain and dural lymphatic vessels.

## Abstract (web summary)

Mechanisms behind molecular transport from cerebrospinal fluid to dural lymphatic vessels remain unknown. This study demonstrates that trans-arachnoid molecular passage does occur and suggests that parasagittal dura may serve as a bridging link between human brain and dural lymphatic vessels.

## Introduction

The human subarachnoid space is shown to be continuous with the paravascular compartment of all brain regions^1^, enabling for cerebrospinal fluid (CSF) to serve as carrier of solutes from brain metabolism into subarachnoid space. Clearance from CSF to blood was for long considered to be dual; one directly to venous blood through arachnoid granulations, and one via lymphatic channels along exiting nerves at the skull base and from the spine^2^. Lymphatic CSF drainage, and the interplay between the lymphatic- and central nervous system (CNS), gained renewed interest when true lymphatic vessels were shown to reside within the dura mater^3,4^. Subsequently, impairment of these meningeal lymphatic pathways has been shown to slow paravascular efflux of amyloid-β from interstitial brain fluid and may possibly represent an aggravating factor in Alzheimer’s disease and age-associated cognitive decline^5^. Neuromodulators from meningeal lymphatics can even possibly modulate brain tissue by their direct release into CSF^6^. A recent animal study was, however, unable to reproduce passage of CSF tracer into dural lymphatic vessels^7^ and was thus in line with previous observations indicating that the arachnoid constitutes an impermeable barrier layer between the CSF and dura mater^8,9^.

Our aim was to explore how these diverging findings of CSF tracer efflux from animal studies translate into a human cohort. We carried out multi-phase magnetic resonance imaging (MRI) for up to 48 h in order to follow the movements of the MRI contrast agent gadobutrol, serving as a CSF tracer. Gadobutrol is a non-ionic, highly hydrophilic molecule of relatively small size (604 Da), prevented by the intact brain–blood–barrier from leakage into CNS blood vessels when administered intrathecally^10^. The substance is therefore suitable for tracing molecular efflux from the cranial compartment along extra-vascular pathways.

## Results

### Patients

The study included 18 patients who were under clinical work-up for possible CSF disorders of various etiologies (Supplementary Table 1).

### CSF tracer movement in CSF spaces

After intrathecal injection of the CSF tracer, its movement over time via foramen magnum and underneath the base of the brain toward the upper medial cerebral convexities is illustrated in Supplementary Movie 1.

### Parasagittal dura

**Fig. 1:**
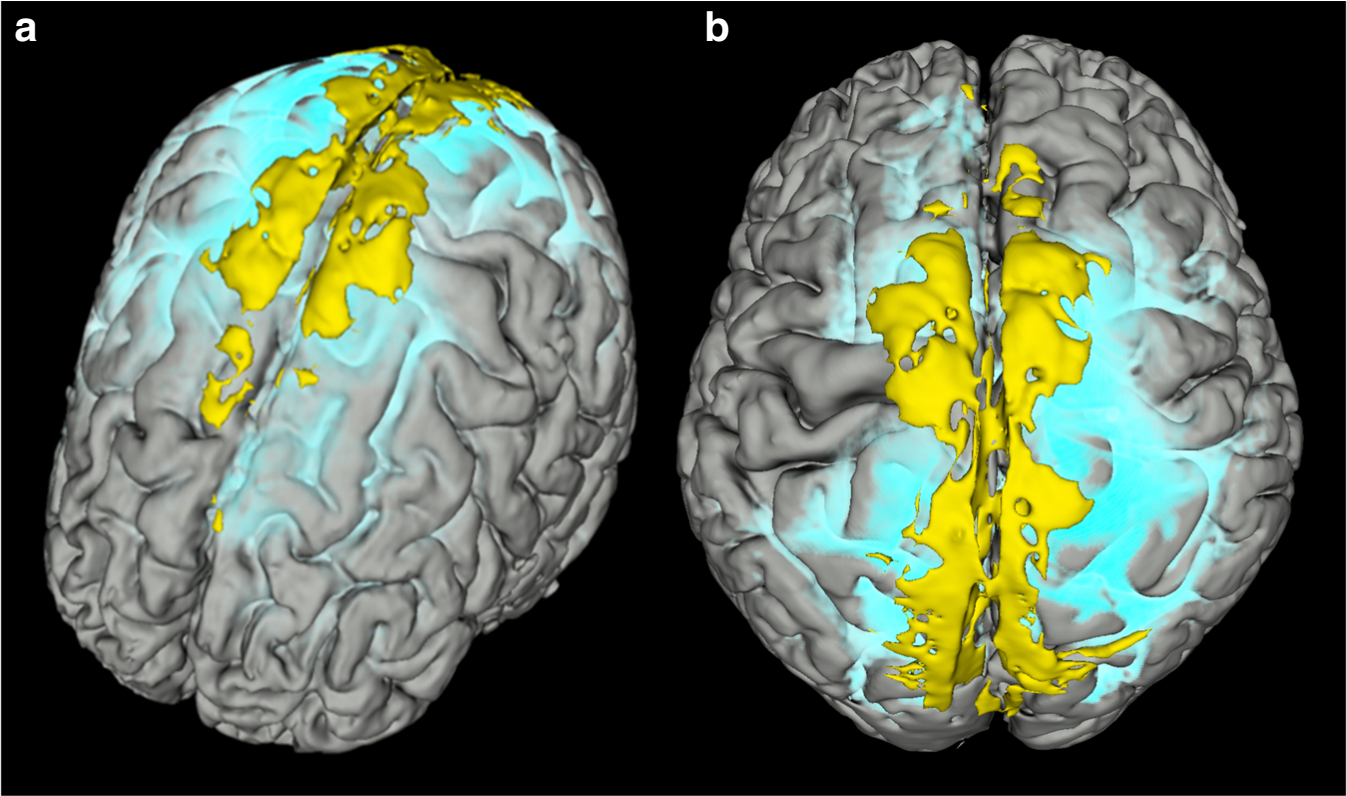
3D representation of parasagittal dura as defined from T2-FLAIR. The brain is shown in oblique coronal view (**a**) and viewed from above (**b**). Parasagittal dura (dark yellow) as defined from T2-FLAIR and co-registered with rough segmentation of the brain (gray) and tracer enhancement in CSF (light blue) from T1 GRE at 48 h after intrathecal administration. The CSF tracer propagated toward the upper brain convexities and parasagittal dura at late time points (at 24 h and particularly at 48 h). A video-animation presenting the full longitudinal extension of parasagittal dura is presented in Supplementary Movie 2. Images: Tomas Sakinis, MD.

Tissue within the parasagittal dura having features as specified above (intermediate or high signal at T2-FLAIR (not free CSF) and at T1-BB (not blood)) was visually identified almost exclusively along the middle to posterior segment of the superior sagittal sinus (Fig. 1; Supplementary Movie 2), corresponding well with the levels where cortical veins enter the sinus.

**Fig. 2:**
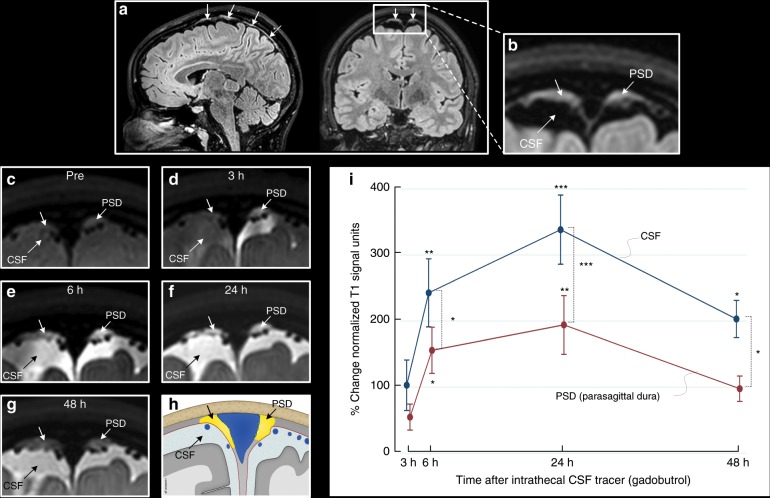
Cerebrospinal fluid (CSF) tracer efflux to parasagittal dura in humans. **a** Sagittal and coronal T2-FLAIR MR images, respectively, show the typical longitudinal and lateral extension of parasagittal dura (PSD) with high signal (arrows) in one study patient. **b** Section of coronal T2-FLAIR demonstrates the lateral extension of PSD (arrows). Similar sections (from T1-BB) reveal change in signal intensity of PSD from before (Pre) CSF tracer injection (**c**), and after 3 h (**d**), 6 h (**e**), 24 h (**f**) and 48 h (**g**). In **h**, the PSD is illustrated in yellow  color (for more details, see Supplementary Fig. 1; illustration: Øystein Horgmo, University of Oslo). The percentage change in normalized T1-BB signal units in CSF (blue line) and PSD (red line) for the entire cohort of 18 individuals is presented in **i**. The signal change was highly significant in both locations (linear mixed model analysis; *P < 0.05, **P < 0.01, ***P < 0.001) and was largest within the CSF. Also note that signal change peaked at 24 h in both CSF and PSD, and signal increase in these structures was highly associated (*R* = 0.92, *P* < 0.001). Error bars refer to 95% confidence intervals (CI).

Even though there were some variations with regard to prominence, tissue in parasagittal dura with features as here defined was depicted in 18/18 study patients and had the typical appearance of longitudinally interspersed areas of up to several millimeters’ transversal width (Fig. 2a, b). After intrathecal injection of CSF tracer, we found enrichment of parasagittal dura at 6, 24 and 48 h and with peak at 24 h (Fig. 2c–h, Tables 1, 2). At group level, there was a highly significant increase in normalized T1 signal units within the parasagittal dura, though more pronounced in nearby CSF (Fig. 2i).

**Table 1:** Information about T1-BB signal units within CSF and ocular bulb.

PatID	T1-BB signal units					T1-BB signal units					Ratio T1-BB signal units					Percentage change from Pre			
	CSF					Ocular bulb					(CSF/ocular bulb)					Normalized T1-BB signal units			
	Pre	3 h	6 h	24 h	48 h	Pre	3 h	6 h	24 h	48 h	Pre	3 h	6 h	24 h	48 h	3 h	6 h	24 h	48 h
1	140	258	334	1009	481	121	83	74	92	124	1.2	3.1	4.5	11.0	3.9	169	290	848	235
2	185	132	151	241	202	114	89	90	113	111	1.6	1.5	1.7	2.1	1.8	−9	3	31	12
3	174	889	572	568	421	114	95	94	125	115	1.5	9.4	6.1	4.5	3.7	513	299	198	140
4	135	–	707	750	403	117	–	72	101	120	1.2	–	9.8	7.4	3.4	–	751	544	191
5	144	83	172	346	257	115	95	94	132	126	1.3	0.9	1.8	2.6	2.0	−30	46	109	63
6	163	213	520	956	574	125	102	98	113	122	1.3	2.1	5.3	8.5	4.7	60	307	549	261
7	160	384	726	889	476	120	95	88	127	115	1.3	4.0	8.3	7.0	4.1	203	519	425	210
8	126	289	618	749	460	98	64	81	85	109	1.3	4.5	7.6	8.8	4.2	251	493	585	228
9	191	164	150	355	440	97	90	65	83	77	2.0	1.8	2.3	4.3	5.7	−7	17	117	190
10	170	466	634	575	326	101	79	76	109	104	1.7	5.9	8.3	5.3	3.1	250	396	213	86
11	156	135	156	647	783	114	86	74	91	120	1.4	1.6	2.1	7.1	6.5	15	54	420	377
12	124	108	205	304	268	93	72	83	114	103	1.3	1.5	2.5	2.7	2.6	13	85	100	95
13	146	133	115	659	756	112	101	89	99	110	1.3	1.3	1.3	6.7	6.9	1	−1	411	427
14	199	191	355	990	788	112	102	92	109	134	1.8	1.9	3.9	9.1	5.9	5	117	411	231
15	177	–	657	947	–	91	–	71	85	–	1.9	–	9.3	11.1	–	–	376	473	–
16	151	110	133	292	363	90	82	86	106	105	1.7	1.3	1.5	2.8	3.5	−20	−8	64	106
17	157	392	610	804	732	107	80	86	110	102	1.5	4.9	7.1	7.3	7.2	234	383	398	389
18	148	114	359	457	506	102	76	72	107	111	1.5	1.5	5.0	4.3	4.6	3	244	194	214
AVG ± STDEV	158 ± 21	254 ± 206	399 ± 229	641 ± 262	484 ± 186	108 ± 11	87 ± 11	83 ± 10	106 ± 15	112 ± 13	1.5 ± 0.3	2.9 ± 2.3	4.9 ± 2.9	6.3 ± 2.8	4.3 ± 1.6	103 ± 152	243 ± 219	338 ± 222	203 ± 116

**Table 2:** T1-BB signal units within parasagittal dura (PSD) and ocular bulb.

PatID	T1-BB signal units					T1-BB signal units					Ratio T1-BB signal units					Percentage change from Pre			
	PSD					Ocular bulb					(PSD/ocular bulb)					Normalized T1-BB signal units			
	Pre	3 h	6 h	24 h	48 h	Pre	3 h	6 h	24 h	48 h	Pre	3 h	6 h	24 h	48 h	3 h	6 h	24 h	48 h
1	176	191	257	1091	519	121	83	74	92	124	1.5	2.3	3.5	11.9	4.2	58	139	715	188
2	176	130	122	197	198	114	89	90	113	111	1.5	1.5	1.4	1.7	1.8	−5	−12	13	16
3	183	454	602	257	168	114	95	94	125	115	1.6	4.8	6.4	2.1	1.5	198	299	28	−9
4	161	–	430	554	328	117	–	72	101	120	1.4	–	6.0	5.5	2.7	–	334	299	99
5	162	102	123	264	226	115	95	94	132	126	1.4	1.1	1.3	2.0	1.8	−24	−7	42	27
6	174	212	668	732	468	125	102	98	113	122	1.4	2.1	6.8	6.5	3.8	49	390	365	176
7	160	255	428	547	372	120	95	88	127	115	1.3	2.7	4.9	4.3	3.2	101	265	223	143
8	143	226	468	634	210	98	64	81	85	109	1.5	3.5	5.8	7.5	1.9	142	296	411	32
9	179	147	149	184	196	97	90	65	83	77	1.8	1.6	2.3	2.2	2.5	−11	24	20	38
10	147	294	528	453	273	101	79	76	109	104	1.5	3.7	6.9	4.2	2.6	156	377	186	80
11	174	142	153	570	613	114	86	74	91	120	1.5	1.7	2.1	6.3	5.1	8	35	310	235
12	135	114	140	192	202	93	72	83	114	103	1.5	1.6	1.7	1.7	2.0	9	16	16	35
13	198	185	143	618	629	112	101	89	99	110	1.8	1.8	1.6	6.2	5.7	4	−9	253	223
14	219	170	262	291	473	112	102	92	109	134	2.0	1.7	2.8	2.7	3.5	−15	46	37	81
15	193	–	366	666	–	91	–	71	85	–	2.1	–	5.2	7.8	–	–	143	269	–
16	146	138	167	147	209	90	82	86	106	105	1.6	1.7	1.9	1.4	2.0	4	20	−15	23
17	151	341	486	474	275	107	80	86	110	102	1.4	4.3	5.7	4.3	2.7	202	300	205	91
18	167	130	296	386	523	102	76	72	107	111	1.6	1.7	4.1	3.6	4.7	4	151	120	188
AVG ± STDEV	169 ± 21	202 ± 95	322 ± 180	459 ± 246	346 ± 159	108 ± 11	87 ± 11	83 ± 10	106 ± 15	112 ± 13	1.6 ± 0.2	2.4 ± 1.1	3.9 ± 2.1	4.5 ± 2.8	3.0 ± 1.3	55 ± 79	156 ± 148	194 ± 188	98 ± 79

**Fig. 3:**
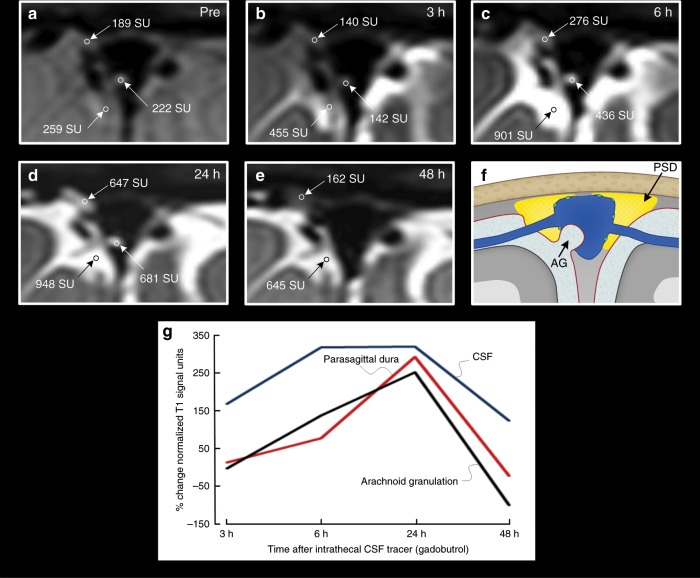
Visualization of the parasagittal dura and an arachnoid granulation. The T1-BB images are shown for one individual (no. 8) prior to intrathecal gadobutrol injection (**a**), and after 3 h (**b**), 6 h (**c**) 24 h (**d**) and 48 h (**e**). The enrichment of CSF tracer is quantified as change in signal units (SU) within the parasagittal dura, CSF space and within one arachnoid granulation. The regions of interest are marked by an open circle. At 48 h (**e**), enhancement of the arachnoid granulation could no longer be detected. In **f** is illustrated the parasagittal dura (PSD, indicated with yellow color) and an arachnoid granulation (AG; illustration: Øystein Horgmo, University of Oslo). In **g**, the percentage change in normalized T1-BB signal units in CSF (blue line), PSD (red line) and arachnoid granulation (black line) in this individual is presented.

As expected, CSF tracer also enhanced in macroscopically visible arachnoid granulations, bulging into the sagittal sinus in scattered locations (Fig. 3). As can be noted, the signal change differed somewhat within the CSF of the subarachnoid space, arachnoid granulations and parasagittal dura, suggesting some level of molecular motion restriction between these structures. Supplementary Movie 3 presents an animation of an arachnoid granulation bulging from parasagittal dura in the superior sagittal sinus.

**Fig. 4:**
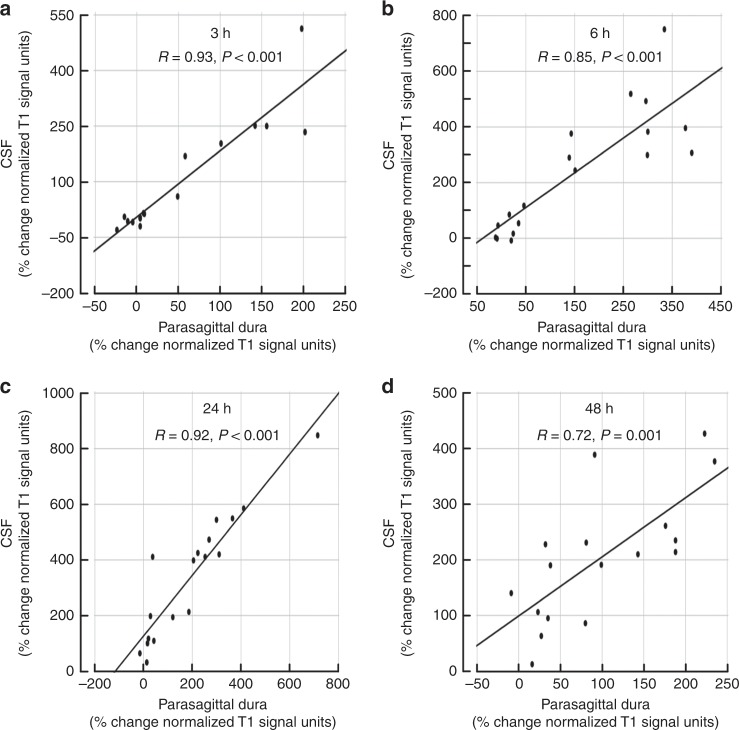
Correlations between CSF tracer enhancement in parasagittal dura and nearby CSF space. There was a highly significant positive correlation between CSF tracer enhancement within parasagittal dura and nearby CSF spaces at 3 h (**a**), 6 h (**b**), 24 h (**c**) and 48 h (**d**). Each plot presents the fit line and the Pearson correlation coefficient (*R*) with *P*-value.

The tracer enrichment in parasagittal dura was highly dependent on presence of tracer in adjacent CSF, which was shown by their strong positive correlation at all time points (3, 6, 24 and 48 h) (Fig. 4). At late scans (i.e. 24 and 48 h, respectively), the remnants of tracer in CSF typically accumulated at the high cerebral convexities and in regions adjacent to parasagittal dura (Fig. 1). Tubular structures with any resemblance to dural lymphatic vessels as previously reported could not be discriminated from other enhancing tissue within parasagittal dura, and may be related to the image resolution of our MRI scans being restricted to 1 × 1 × 1 mm voxel size.

**Fig. 5:**
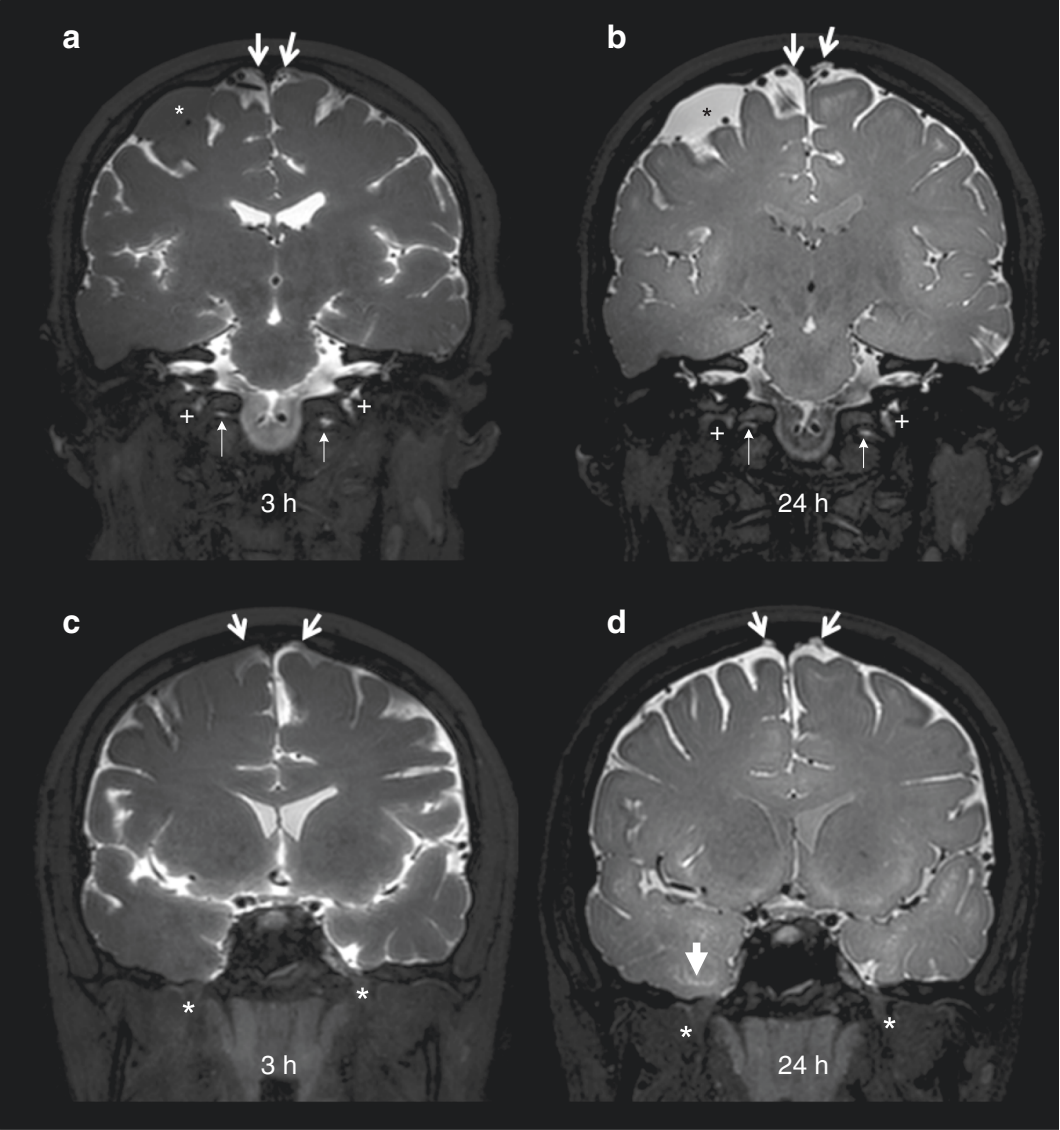
Evidence for CSF efflux along cranial nerves. Posterior (**a**, **b**) and anterior (**c**, **d**) mid-coronal T1-BB from a patient (no. 10) under work-up for symptoms possibly related to an arachnoid cyst (asterisks in **a** and **b**, respectively) at the upper convexity of the right cerebral hemisphere. As compared to 3 h after intrathecal gadobutrol (**a**), the cyst enhanced homogenously at scans after 24 h (**b**). Enrichment of parasagittal dura (thick arrows) increased from 3 (**a**, **c**) to 24 h (**b**, **d**), respectively. CSF tracer within brain perivascular spaces is well illustrated with T1-BB MRI (exemplified as pointed out by arrowheads; **d**). In addition is shown CSF tracer enhancement below the skull base outlets of the jugular foramen (crosses) and hypoglossal canal (thin arrows) (**a**, **b**). In the same patient, there was no sign of tracer enhancement along the mandibular nerve below level of the oval foramen (asterisks) at any time point (**c**, **d**).

For the visual assessment of tracer enhancement on T1-BB below selected cranial nerve outlets at the skull base, this was detected below the hypoglossal canal in 17/18, the jugular foramen in 17/18, the cribriform plate in 2/18, and the foramen ovale in 0/18 individuals (Fig. 5).

## Discussion

This study demonstrates for the first time direct efflux of a CSF tracer to parasagittal dura in humans. The findings render for the parasagittal dura to represent a bridging link for CSF-mediated exchange of molecules between brain tissue and dural lymphatic vessels.

**Fig. 6:**
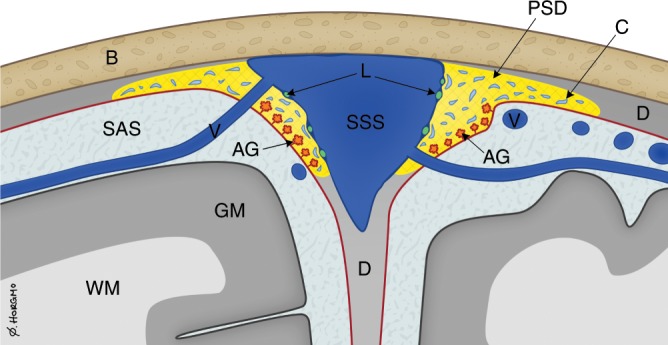
Cartoon illustrating reported microstructural elements within the parasagittal dura based on refs. ^14,15^. **a** The parasagittal dura (PSD, colored yellow) contain a network of intradural channels (C, colored light blue), coalescing into lateral lacunae medially (not shown), being in close relation with a dense carpet of intradural arachnoid granulations (AG, orange color). PSD is most prominent at level where the cortical veins (V, dark blue) enter the superior sagittal sinus (SSS, dark blue). Lymphatic vessels (L, green) reside at the SSS wall. The microstructural elements within PSD are of sub-millimeter thickness and therefore not expected to be visualized on MRI with 1 mm resolution. D dura, GM gray matter, SAS subarachnoid space, WM white matter. Illustrations: Øystein Horgmo, University of Oslo.

The extent of enhancing tissue within parasagittal dura does not resemble the previously reported appearance of true dural lymphatic vessels, which are tubular-shaped structures of sub-millimeter cross-sectional diameter near the venous sinus wall^3,4,11^, and also far from the classical MRI appearance of arachnoid granulations bulging into venous sinuses^12^. Our findings compare better with previous anatomical reports demonstrating that parasagittal dura along the middle one-third of the superior sagittal sinus harbors a dense carpet of arachnoid granulations medially, being in close association with an extensive network of intradural channels from 0.02 to 2.0 mm in diameter, coursing and coalescing into lateral lacunae from laterally toward the superior sagittal sinus^13,14^ (illustrated in Fig. 6). The endothelium of intradural channels was proposed to possibly be the final barrier for the absorption of CSF to venous blood^15^, but passage of CSF over this cell layer has never since been confirmed in vivo. The in vivo-based observations from the current MRI study show that parasagittal dura does not drain CSF only, but also molecules, with high level of dependence on their presence in the adjacent subarachnoid compartment. In light of more recent discoveries showing evidence of true dural lymphatic vessels in the superior sagittal sinus wall^3,4^, and the fact that lymphatic vessels as well as blood vessels within the dura lack tight junctions^16^, it seems plausible that molecules within parasagittal dura structures may well reach adjacent lymphatic vessels, and perhaps also vice versa. The parasagittal dura may thus represent a bridging link for CSF-mediated exchange of molecules between brain tissue and dural lymphatic vessels.

The region with tracer enhancement, as presented here, stretches laterally to a larger extent than what can be anticipated from the previous anatomical descriptions of intradural human arachnoid granulations^14,15^, which should be highly suggestive for tracer escape to dura outside arachnoid granulations and into the network of intradural channels. To this end, we note that any CSF or molecular escape to arachnoid granulations whatsoever has lately been questioned^7^, and explained by commonly accepted assumption of the impermeable arachnoid-barrier.

Tracer enrichment in parasagittal dura is furthermore conflicting evidence against a recent animal study, where basal meningeal lymphatic vessels were proposed being more important than dorsal (parasinusoidal) lymphatic vessels for CSF molecular uptake and transport due to their closer proximity to the subarachnoid space in areas with a much thinner dural layer^17^. Even though we also observed signs of lymphatic drainage through basal neuroforamina, the observations do not strengthen the hypothesis that the importance of molecular drainage toward parasagittal structures is inferior to basal outflow. The very thin, linear structures of tracer enhancement we observed below the scull base, and our image resolution with voxel size 1 mm, did not allow us to quantify the relative importance between parasagittal and basal outflow pathways. While our study corroborates findings from animal studies by showing that CSF molecular outflow through skull base foramina occurs also in humans, the presumed importance of the transnasal lymphatic route^18^ requires further exploration in humans. In this study, signs of tracer enhancement below the cribriform plate could be visually confirmed in only 2/18 patients. The olfactory system is indeed of proportionally much lesser size in humans than in rodents, potentially suggesting this molecular clearance route is of less importance, at least relative to clearance toward other cranial nerve outlets at the skull base and the parasagittal dura as corroborated by the current observations.

The present findings also stand in contradistinction to a recent CSF tracer study of mice reporting no signs of tracer propagation through outside the arachnoid layer, and concluding that CSF outflow occurred predominately along cranial nerves through skull neuroforamina^7^. With concern to clearance rate, another rodent study reported very quick exit of tracer from CSF along perineural routes at the skull base^19^. In our human cohort, tracer was present in CSF at a far longer time span (even after 2 days), and the tail of the remaining tracer bolus at late scans along parasagittal dura (Fig. 1; Supplementary Movie 1) may also indicate a net drift of CSF molecules in this direction, rather than toward the skull base. These obvious discrepancies between observations in mice and humans underline the importance of human translational research.

It has previously been shown that neurotoxic molecules such as amyloid-β and tau are cleared from brain tissue along paravenous pathways (the glymphatic system)^20^ and speculated that molecules also drain further along cerebral veins along the brain convexities into dural lymphatic vessels^4^. A MRI study suggested the same based on peak CSF tracer enhancement in brain and cervical lymph nodes occurring at the same time point^21^. It has, however, been demonstrated in rats that from the total amount of MRI contrast agent injected into the cisterna magna, merely 19% enriched the brain parenchyma, the remainder escaped via other routes^22^. Although the dose, injection site and species differences may be important for this fraction, the present observations of high association between tracer enhancement in CSF and adjacent parasagittal dura suggest that molecules within the CSF primarily drain directly to lymphatic pathways. The extent to which this molecular flux may contribute to brain parenchyma clearance remains to be investigated. In this context, previous findings showing that impairment of meningeal lymphatic function slows paravascular influx of macromolecules into the brain, as well as efflux from the interstitial space^5^, may suggest that impaired lymphatic clearance function may exert its negative effect directly on the accumulation of molecules within the CSF, and thereby further upstream on molecular exit from brain into CSF.

An obvious limitation of this study is the MR image resolution of 1 mm voxel size. The possible interchange between microscopic contents of the parasagittal dura, such as intradural channels and arachnoid granulations, and dural lymphatic vessels, should be further explored in future studies. In the lack of T1-mapping techniques with image resolution even close to 1 mm, we did not have the opportunity to go beyond semi-quantitative measures of tracer enrichment. Ideally, we could also have used an extra-cranial device as reference for normalization of signal units, as the ocular bulbs may be affected by some motion artifacts during scans of several minutes. Moreover, the long duration of molecular clearance from human CSF, and the high demand on patients and resources associated with multi-phase MR imaging over 48 h, has limited the number of scans we were able to perform in single patients through this time span. Therefore, the time point for peak tracer enhancement in human parasagittal dura must be considered to represent a rough estimate.

In conclusion, the present study demonstrates in vivo escape of a CSF tracer from the subarachnoid compartment over the arachnoid and into the parasagittal dura along the superior sagittal sinus in humans. The parasagittal dura may thus represent a bridging link for CSF-mediated exchange of molecules between brain tissue and dural lymphatic vessels.

## Methods

### Ethical permissions

The study was approved by The Institutional Review Board (2015/1868), Regional Ethics Committee (2015/96) and the National Medicines Agency (15/04932-7). Patients were included after written and oral informed consent. The study was registered in Oslo University Hospital Research Registry (ePhorte 2015/1868).

### Experimental design

The study design was prospective and observational: randomization of patients was not relevant; neither was a priori sample size calculation needed.

### Patients

The study included consecutive individuals referred to the Department of neurosurgery, Oslo University Hospital—Rikshospitalet, Oslo, Norway, for various CSF circulation disorders (Supplementary Table 1). Exclusion criteria were: history of hypersensitivity reactions to contrast media agents, history of severe allergy reactions in general, evidence of renal dysfunction (i.e. normal glomerular filtration rate, GFR), age <18 or >80 years, pregnant or breastfeeding women.

### MRI protocol

Scans were obtained in a 3-Tesla MRI unit (Philips Ingenia®) with a 32-channel head coil.

1. Whole-brain 3D T2-Fluid Attenuated Inversion Recovery (FLAIR), repetition time (TR)/echo time (TE)/inversion time (TI) = 4800/311/1650 ms, echo train length = 167, flip angle = 90°, 2 averages, 1 × 1 × 1 mm voxel size (isotropic), acquisition time = 5 min and 41 s.
2. Whole-brain 3D T1-Black-blood (BB) scan with TR/TE = 700/35 ms, echo train length = 55, flip angle = 80°, 2 averages, 1 × 1 × 1 mm voxel size (isotropic) and acquisition time 4 min and 54 s.
3. Whole-brain 3D T1-gradient echo (GRE) with TR = shortest (typically 5.1 ms), echo time = shortest (typically 2.3 ms), echo train length = 232, flip angle = 8°, 1 average, 1 × 1 × 1 mm voxel size and acquisition time 6 min and 29 s.

We repeated the T1-weighted scans at 3, 6, 24 and 48 h, respectively, after intrathecal injection of 0.5 ml gadobutrol (1 mmol/ml) (Gadovist®, Bayer AB, Sweden). Gadobutrol injection was preceded by injection of 3 ml of 270 mg I/ml iodixanol (Visipaque®, GE Healthcare, Norway) to ensure correct needle position within the intrathecal compartment. Patients were kept in the supine position with a pillow under their heads until the 6 h scan was completed and were then allowed to move freely.

### Image analysis

Sagittal T2-fluid FLAIR, as well as T1-BB scans from all time points, were reformatted into coronal and axial 1-mm slices perpendicular and parallel to the plane defined by a line between the anterior and posterior commissures (AC–PC plane), respectively. Identically positioned coronal slices were used to identify parasagittal dura with tissue characteristics remaining unsuppressed (intermediate or high signal) at both T2-FLAIR and T1-BB. T2-FLAIR has the ability to suppress free (unbound) CSF within the ventricular compartment and subarachnoid space^23^. T1-BB images are tuned to darken the contents of blood vessels, even when containing a contrast agent^24^. Thus, in areas with moving protons, such as in arteries and veins, the T1-BB signal is nulled. Around the high brain convexities, there are little motions within the CSF, and therefore the signal is not black (nulled) as in the blood vessels, rendering for a more intermediate CSF-signal.

In areas of parasagittal dura with these features, regions of interest (ROIs) were placed in identical locations at all time points and well within the outer perimeter of parasagittal dura at T1-BB images for robust measurement of T1 signal change as a sign of tracer enrichment. In addition, a ROI was placed within adjacent subarachnoid space to assess T1 signal change in CSF. The CSF tracer enrichment was measured as change in T1-BB signal units, and was normalized against the reference (i.e. the vitreous body of the ocular bulb) to correct for any baseline shift of image grayscale between time points (Supplementary Fig. 1). We also visually assessed any presence of tracer enhancement on T1-BB images below selected cranial nerve outlets at the skull base and dichotomized this as yes/no whether such enhancement was found present at any time point, or not, respectively.

3D T1-GRE scans were used for segmentation and co-registration rendering for 3D representations (using 3D Slicer version 4 and SPM 12, both open source software) illustrating the relations between the brain surface and CSF tracer enhancement. T1-GRE was, however, unsuitable for precise ROI-placement in parasagittal dura, since the border against the enhancing CSF compartment became effaced at late scans (Supplementary Fig. 2).

### Statistics

Statistical analyses were performed using the SPSS software version 22 (IBM Corporation, Armonk, NY). Differences between continuous data were determined using linear mixed models with a random intercept. Correlations were determined by Pearson correlation coefficient. Statistical significance was accepted at the 0.05 level (two-tailed).

### Reporting summary

Further information on research design is available in the Nature Research Reporting Summary linked to this article.

## Supplementary information

Supplementary Information

Peer Review

Reporting Summary

Description of Additional Supplementary Files

Supplementary Movie 1

Supplementary Movie 2

Supplementary Movie 3

## Data Availability

The data presented in this work are available upon request.
