# Supplementary material for: Cerebrospinal fluid tracer efflux to parasagittal dura in humans
Source: Nat Commun. 2020 Jan 17;11:354. doi: 10.1038/s41467-019-14195-x (PMC6969040; doi:10.1038/s41467-019-14195-x)
Supplement: Supplementary file 1 — Supplementary Information [file 41467_2019_14195_MOESM1_ESM.pdf]

Supplementary Information

Cerebrospinal fluid tracer efflux to parasagittal dura in humans

Geir Ringstad[a], Per Kristian Eide[b,c]

[a]*Dept. of Radiology and Nuclear Medicine, Oslo University Hospital - Rikshospitalet,*

[b]*Dept. of Neurosurgery, Oslo University Hospital-Rikshospitalet,* [c]*Institute of Clinical Medicine, Faculty of Medicine, University of Oslo, Oslo, Norway*

Correspondence:

Professor Per Kristian Eide, MD PhD

Department of Neurosurgery

Oslo University Hospital - Rikshospitalet

Pb 4950 Nydalen,

N-0424 Oslo, Norway

p.k.eide@medisin.uio.no/peide@ous-hf.no

Supplementary Table 1. Patients

PatID	Diagnosis	Age (Years)	Gender F	Gender M	BMI (kg/m2)
**1**	Arachnoid cyst	44	1		34.1
**2**	Idiopathic intracranial hypertension	33	1		19.6
**3**	Pineal cyst	25	1		31.1
**4**	Pineal cyst	29	1		23.3
**5**	Idiopathic intracranial hypertension	26	1		21.2
**6**	Idiopathic intracranial hypertension	38	1		30.1
**7**	Suspected intracranial hypotension*	34	1		17.6
**8**	Idiopathic intracranial hypertension	30	1		30.5
**9**	Arachnoid cyst	58		1	25.1
**10**	Arachnoid cyst	20	1		30.9
**11**	Suspected intracranial hypotension*	52		1	26.8
**12**	Idiopathic intracranial hypertension	23	1		24.1
**13**	Arachnoid cyst	75		1	24.6
**14**	Suspected intracranial hypotension*	27		1	18.6
**15**	Idiopathic intracranial hypertension	51		1	32.8
**16**	Idiopathic intracranial hypertension	28	1		28.4
**17**	Idiopathic intracranial hypertension	37	1		35.4
**18**	Arachnoid cyst	70	1		27.1
**AVG ± STDEV**		**39 ± 16**			**27 ± 5**

*No CSF leakage was verified in the three individuals with suspected intracranial hypotension.

The patient material included 18 individuals that underwent MRI including T1-BB for the tentative diagnoses arachnoid cyst (n=5), idiopathic intracranial hypertension (n=8), pineal cyst (n=2), and suspected intracranial hypotension (n=3). None had previously undergone surgery. Their average age was 39 ± 16years.

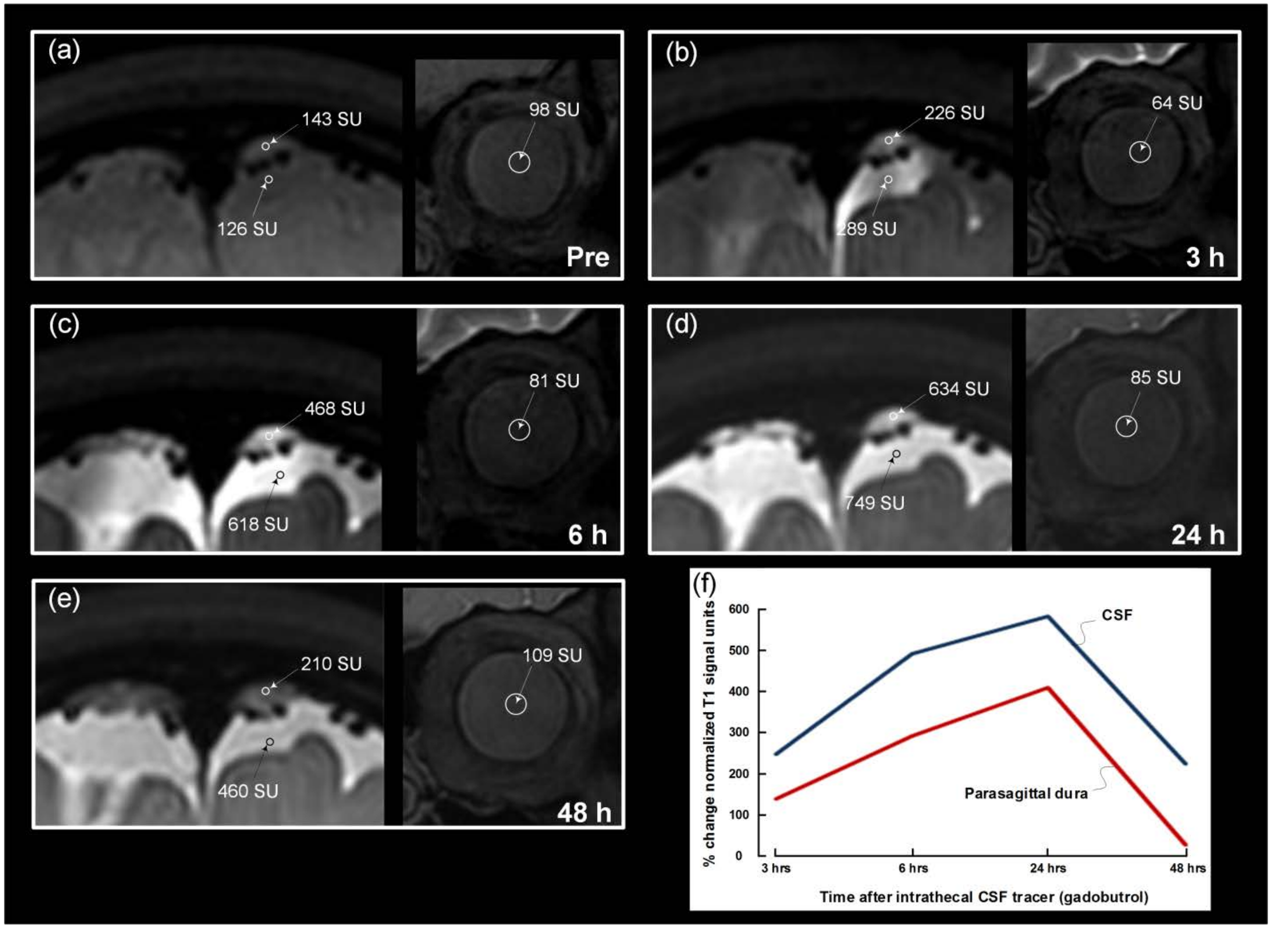

Supplementary Figure 1. Normalization of signal unit change on T1-BB in parasagittal dura against reference tissue (ocular bulb). Details from coronal sections are shown for one individual (no 8) prior to intrathecal gadobutrol injection (a), and after 3 h (b), 6 h (c) 24 h (d) and 48 h (e). For each time point, the left image shows the parasagittal dura and nearby subarachnoid space; the reference location (i.e. the vitreous body of the ocular bulb) is shown to the right. The enrichment of CSF tracer is assessed as change in signal units (SU) within the regions of interest that are marked by an open circle. The measured signal units were normalized against the reference to correct for any baseline shift of image greyscale between time points. The changes in signal units and percentage change based on normalized values (f) for each individual included in the study are given in Tables 1 and 2.

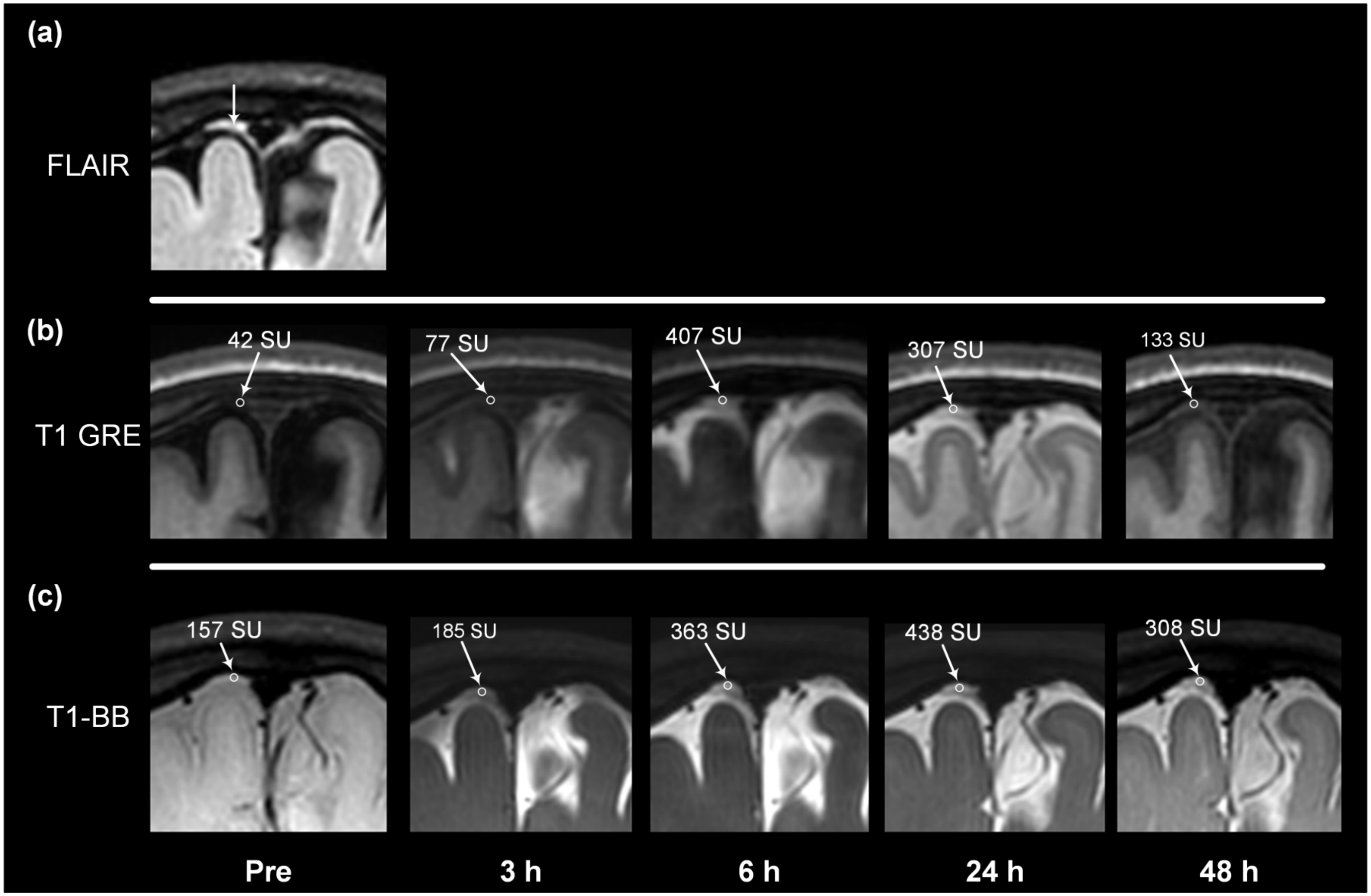

Supplementary Figure 2. T1 GRE from individual 10. (a) The coronal T2-FLAIR MR image shows the transversal extension of parasagittal dura (PSD) with high signal (arrows). The T1 GRE (b) and T1-BB (c) are shown before (Pre) intrathecal administration of gadobutrol, and after 3, 6, 24 and 48 hours, increased signal as sign of tracer enhancement within the PSD for both T1 GRE and T1-BB. As T1-BB images in general were best suited for depiction of the PSD outer boundaries at different time points, allowing for more robust measurements, these were therefore used in the ROI analysis.
