# Supplementary material for: Cerebrospinal fluid tracer efflux to parasagittal dura in humans
Source: Nat Commun. 2020 Jan 17;11:354. doi: 10.1038/s41467-019-14195-x (PMC6969040; doi:10.1038/s41467-019-14195-x)
Supplement: Supplementary file 3 — Reporting Summary [file 41467_2019_14195_MOESM3_ESM.pdf]

nature research

Corresponding author(s): Per Kristian Eide

Last updated by author(s): Dec 10, 2019

Reporting Summary

Nature Research wishes to improve the reproducibility of the work that we publish. This form provides structure for consistency and transparency in reporting. For further information on Nature Research policies, see Authors & Referees and the Editorial Policy Checklist.

Statistics

For all statistical analyses, confirm that the following items are present in the figure legend, table legend, main text, or Methods section.

n/a	Confirmed	
☐	☒	The exact sample size (*n*) for each experimental group/condition, given as a discrete number and unit of measurement
☐	☒	A statement on whether measurements were taken from distinct samples or whether the same sample was measured repeatedly
☐	☒	The statistical test(s) used AND whether they are one- or two-sided<br>*Only common tests should be described solely by name; describe more complex techniques in the Methods section.*
☐	☒	A description of all covariates tested
☒	☐	A description of any assumptions or corrections, such as tests of normality and adjustment for multiple comparisons
☐	☒	A full description of the statistical parameters including central tendency (e.g. means) or other basic estimates (e.g. regression coefficient) AND variation (e.g. standard deviation) or associated estimates of uncertainty (e.g. confidence intervals)
☐	☒	For null hypothesis testing, the test statistic (e.g. *F*, *t*, *r*) with confidence intervals, effect sizes, degrees of freedom and *P* value noted<br>*Give P values as exact values whenever suitable.*
☒	☐	For Bayesian analysis, information on the choice of priors and Markov chain Monte Carlo settings
☒	☐	For hierarchical and complex designs, identification of the appropriate level for tests and full reporting of outcomes
☐	☒	Estimates of effect sizes (e.g. Cohen's *d*, Pearson's *r*), indicating how they were calculated

Our web collection on statistics for biologists contains articles on many of the points above.

Software and code

Policy information about availability of computer code

Data collection	Performed in the hospital picture archiving and communication system (PACS): SECTRA IDS7 (SECTRA, Sweden)
Data analysis	SPSS software version 22 (IBM Corporation, Armonk, NY)

For manuscripts utilizing custom algorithms or software that are central to the research but not yet described in published literature, software must be made available to editors/reviewers. We strongly encourage code deposition in a community repository (e.g. GitHub). See the Nature Research guidelines for submitting code & software for further information.

Data

Policy information about availability of data

All manuscripts must include a data availability statement. This statement should provide the following information, where applicable:

- Accession codes, unique identifiers, or web links for publicly available datasets
- A list of figures that have associated raw data
- A description of any restrictions on data availability

The source data are available in Supplementary material, and anonymized images may be provided on request. The authors have access to all data used in the study.

Field-specific reporting

Please select the one below that is the best fit for your research. If you are not sure, read the appropriate sections before making your selection.

☒ Life sciences ☐ Behavioural & social sciences ☐ Ecological, evolutionary & environmental sciences

Life sciences study design

All studies must disclose on these points even when the disclosure is negative.

Sample size	No sample-size calculation was performed. The sample size was 18 patients. The available number of patients is limited, as intrathecal MRI contrast agent injections are performed off-label and by special permission from the National Medicines Agency of Norway.
Data exclusions	We excluded patients who were diagnosed with CSF leaks. This criteria was pre-established, as CSF hypotension may considerable affect CSF tracer enrichment.
Replication	In this human study, performing off-label intrathecal injections of contrast agent, the studies could for ethical reasons not be carried out more than one time and were therefore not replicated.
Randomization	We used an observational study design, the issue of randomization is therefore not relevant here.
Blinding	The investigator who performed the image analysis was blinded to clinical diagnosis.

Reporting for specific materials, systems and methods

We require information from authors about some types of materials, experimental systems and methods used in many studies. Here, indicate whether each material, system or method listed is relevant to your study. If you are not sure if a list item applies to your research, read the appropriate section before selecting a response.

Materials & experimental systems

n/a	Involved in the study	
☒	☐	Antibodies
☒	☐	Eukaryotic cell lines
☒	☐	Palaeontology
☒	☐	Animals and other organisms
☐	☒	Human research participants
☐	☒	Clinical data

Methods

n/a	Involved in the study	
☒	☐	ChIP-seq
☒	☐	Flow cytometry
☐	☒	MRI-based neuroimaging

Human research participants

Policy information about studies involving human research participants

Population characteristics	We investigated consecutive patients referred to our neurosurgical department for work-up of various cerebrospinal fluid (CSF) circulation disorders, including arachnoid cysts, pineal cysts, idiopathic intracranial hypertension and suspected intracranial hypotension (age was 39±16 (mean±stdev) years, 13 female, 5 men).
Recruitment	Study participants were recruited consecutively as they were referred to the neurosurgical department for work-up of various CSF circulation disorders. We identify no selection biases.
Ethics oversight	The study was approved by The Institutional Review Board (2015/1868), Regional Ethics Committee (2015/96) and the National Medicines Agency (15/04932-7). Patients were included after written and oral informed consent.

Note that full information on the approval of the study protocol must also be provided in the manuscript.

Clinical data

Policy information about clinical studies

All manuscripts should comply with the ICMJE guidelines for publication of clinical research and a completed CONSORT checklist must be included with all submissions.

Clinical trial registration	This was an observational study and no interventional study; the study was therefore not registered in clinicaltrials.org.
Study protocol	The study is registered in the Oslo university hospital research registry: ePhorte 2015/1868.
Data collection	Study patients were investigated with MRI at the Interventional Centre at Oslo University Hospital, Oslo, Norway, as part of their general work-up for suspicion of various CSF circulation disorders at the Department of Neurosurgery at Oslo University Hospital, Norway. Time period of recruitment was from April-December 2018 and the study period was from June 2018 to February 2019.
Outcomes	Predefined primary outcomes: CSF tracer dependent MRI signal change in parasagittal dura. Predefined secondary outcome: CSF tracer dependent signal increase at selected cranial nerve outlets at the skull base.

Magnetic resonance imaging

Experimental design

Design type	The study did not utilize fMRI methods, neither resting state or task, nor event-related or block design.
Design specifications	This was not a fMRI study.
Behavioral performance measures	This was not a fMRI study.

Acquisition

Imaging type(s)	Structural MRI sequences that was used in this study: T1-Black Blood and T2-FLAIR. No functional studies (fMRI) involved.
Field strength	3 Tesla
Sequence & imaging parameters	T1-Black Blood: TR/TE = 700/35 ms, echo train length = 55, flip angle = 80 degrees, 2 averages, 1x1x1 mm voxel size (isotropic) and acquisition time 4 minutes and 54 seconds<br>T2-FLAIR: TR/TE/TI = 4800/311/1650 ms, echo train length = 167, flip angle = 90 degrees, 2 averages, 1x1x1 mm voxel size (isotropic), acquisition time = 5 minutes and 41 seconds
Area of acquisition	Whole brain and cranium.

Diffusion MRI ☐ Used ☒ Not used

Preprocessing

Preprocessing software	No preprocessing performed, MR images were directly analyzed in the hospital PACS.
Normalization	MRI signal units measured at area of interest (parasagittal dura) were normalized to reference tissue (ocular bulb) to correct for any change in greyscale baseline.
Normalization template	No template used.
Noise and artifact removal	None used.
Volume censoring	Not performed.

Statistical modeling & inference

Model type and settings	Differences between continuous data were determined using linear mixed models with a random intercept. Correlations were determined by Pearson correlation coefficient. Statistical significance was accepted at the .05 level (two-tailed).
Effect(s) tested	We studied tracer enrichment in CSF and parasagittal dura by ROI measurement of normalized MRI signal unit change and also visually assessed for contrast enhancement (yes/no) at selected cranial nerve outlets at the skull base.

Specify type of analysis: ☐ Whole brain ☐ ROI-based ☒ Both

Anatomical location(s): Anatomical locations were selected by visual inspection (not automatically) and assessed in part by ROI analysis (parasagittal dura) and (visually at selected cranial nerve root outlets).

Statistic type for inference (See Eklund et al. 2016)	Not relevant, voxel- or clusterwise methods were not applied.
Correction	Multiple comparisons were not performed.

Models & analysis

n/a	Involved in the study
☒	☐ Functional and/or effective connectivity
☒	☐ Graph analysis
☒	☐ Multivariate modeling or predictive analysis
