# Supplementary material for: Cerebrospinal fluid tracer efflux to parasagittal dura in humans
Source: Nat Commun. 2020 Jan 17;11:354. doi: 10.1038/s41467-019-14195-x (PMC6969040; doi:10.1038/s41467-019-14195-x)
Supplement: Supplementary file 4 — Description of Additional Supplementary Files [file 41467_2019_14195_MOESM4_ESM.docx]

**Description of Additional Supplementary Files**

File name: Supplementary Movie 1
Description: CSF tracer distribution following intrathecal administration.
Volume rendered (VR) T1 GRE MRI with rough segmentation of the brain demonstrating peak tracer enhancement in CSF outside the upper brain convexities at 24 hours (in light blue), co-registered with parasagittal dura from FLAIR-images (in dark yellow). At late time points (24 and 48 hours, respectively), the tracer had after its early entry at the foramen magnum migrated from underneath the base of the brain towards the upper medial cerebral convexities adjacent to the parasagittal dura, particularly at 48 hours. Video: Tomas Sakinis, MD.

File name: Supplementary Movie 2
Description: 3D representation of parasagittal dura.
Parasagittal dura (dark yellow) was defined from T2-FLAIR and co-registered with rough segmentation of the brain (Video: Tomas Sakinis, MD).

File name: Supplementary Movie 3
Description: 3D representation of an arachnoid granulation and parasagittal dura (overlay on T1-BB slice for anatomical overview).
The enhancing arachnoid granulation segmented from T1-BB at 24 hours (in orange color) bulges into the superior sagittal sinus (blue) from the inferior part of parasagittal dura (from co-registered FLAIR, dark yellow) (Video: Tomas Sakinis, MD). Intradural arachnoid granulations within parasagittal dura, as reported in (1), are not depicted at MRI, which is limited by an image resolution of 1x1x1 mm (voxel size).

1. Fox RJ, Walji AH, Mielke B, et al. Anatomic details of intradural channels in the parasagittal dura: a possible pathway for flow of cerebrospinal fluid. Neurosurgery. 1996;39(1):84-90; discussion -1.
