# Supplementary material for: Cerebrospinal fluid tracer efflux to parasagittal dura in humans
Source: Nat Commun. 2020 Jan 17;11:354. doi: 10.1038/s41467-019-14195-x (PMC6969040; doi:10.1038/s41467-019-14195-x)
Supplement: Supplementary file 2 — Peer Review [file 41467_2019_14195_MOESM2_ESM.pdf]

Reviewers' comments:

Reviewer #1 (Remarks to the Author):

Ringstad and Eide apply multi-phase MRI in combination with intrathecal gadobutrol in human subjects and report on a tissue compartment along the superior sagittal sinus a.k.a. “parasagittal dura, PSD” identified on sagittal T2-FLAIR MRIs in 18 subjects.

Major findings and conclusions:
• In human subjects the PSD was identified as a compartment with the following characteristics: 1) intermediate to high signal intensity on T2-FLAIR images after intrathecal gadobutrol; 2) normalized signal intensity significantly higher than adjacent CSF in subarachnoid space on T1-weighted (‘black blood’, BB) images at 6, 24 and 48 hrs after intrathecal contrast; and 3) the PSD is not intravascular because the BB or blood nulling T1 sequence did not void it out
• Signal changes in the PSD was found to be highly correlated with signal in adjacent subarachnoid space at each timepoint after intrathecal contrast delivery
• Signal changes in one identified Pacchionian granulation protruding into the dural sinus was also captured with what appears to be a similar time course (peak at 24 hrs).
• Authentic dural lymphatics could not be identified
• The authors that ‘we demonstrate trans-arachnoid tracer escape from CSF in man”. They further speculate that “this observation suggest that molecules drain directly from CSF to parasagittal dura and not primarily along veins exiting the brain”

Comments:
The identification of the PSD based on multiphase MRI and intrathecal contrast is indeed novel and intriguing especially since this is an observation in live human brain. The question remains however, what this compartment really represents? i.e. does it truly ‘belong’ to dura? and/or does it represent a solute draining route as claimed by the authors? Could it be a collection of microscopic arachnoid villi (which cannot be captured at the spatial resolution)? or is it an artifact due to incomplete blood suppression? Answers to these questions are clearly required for further interpretation.

To this reviewer the most important question in regards to the PSD compartment is whether or not it truly represents a draining route i.e. there is no concrete evidence of this in the presented data.

The time course of signal changes captured in the subarachnoid CSF, PSD and Pacchionian granulation are concurrent – it would be important to have the normalized signal changes in the pacchioninan granulation for comparison.

The T1 weighted BB sequence can be challenging and signal suppression within the vessel lumen is largely dependent on fast arterial blood flow. Because of this dependency, the images are highly susceptible to flow-related artifacts – could the PSD changes be influenced by incomplete blood suppression?

More examples from other subjects of PSD anatomy would be important and informative. Any further volumetric quantification of the PSD compartment in relation to dura is lacking.

Reviewer #2 (Remarks to the Author):

This manuscript is a follow up on the Oslo team explorations of CSF transport in the human brain and surrounding structure. The authors collect repeated scans over 48 hrs after intrathecal delivery of a contrast agent in 18 patients. They report that the contrast agent accumulates in the parasagittal dura. This conclusion is based on clever use of T2-FLAIR, which suppress the signal from the free fluid filled compartments such as the subarachnoid space, whereas T1-BB images darken the contents of blood vessels. The authors define tissue with an intermediate T2-FLAIR signal that are not blood vessels as dura. This tissue with these imaging characteristics was detected almost exclusively along with the middle third of the superior sagittal sinus. The imaging does very convincingly show that the contrast agent accumulates in this space. My major concern is that the evidence for this is a

compartment of dura is indirect. Why not image a cadaver and then follow the imaging up with traditional histology? Albeit I am not an expert, the compartment with retention of the contrast agent is huge compared with the sinus and does not look like the classical cross section of dura in medical textbooks. The compartment might be a specialization of the subarachnoid space containing multiple membranes that subdivide the space and reduce the T2-FLAIR. Also, it seems odd that dura which is separated from the subarachnoid space by a barrier of tight junctions would take up and retain so large quantities of the contrast agent.

Reviewer #3 (Remarks to the Author):

In the manuscript "Cerebrospinal fluid tracer efflux to parasagittal dura in humans" the authors describe experiments to demonstrate the clearance of CSF from the brain using MRI contrast agent (Gad) introduced into the CSF compartment in humans. Follow-up for 2 days show enhancement of the parasinal dura (PSD), which peaked at about one day after injection of the Gad. Experiments such as the one described here is critical in better understanding of CSF clearance and lymphatic access, which may have implications in many diseases. However, a few clarifications are required before the article can be accepted:

1.The CSF is not black in the pre-Gad T1 black-blood (BB) images (Fig 1c, suppl fig 1a, suppl fig 2a), indicating that these might be more like a PD image. Please clarify.
2.The pulse sequences used in the manuscript is limited to FLAIR and T1BB. It would have been immensely useful to have a T1-map or even a GRE T1-weighted image to quantify these changes. It might have been possible to (at least partially) quantify the GAD in the sagittal sinuses using GRE sequence.
3.Along these lines, normalization with vitreous signal in the eye was a good idea for lineally changing signal. However, this may not be the case here as scans were performed in multiple sessions, especially with dramatic contrast changes in the brain. It is conceivable that there are non-linear effects from B1 and B0 shimming, that affect the ocular region differently than the region of the sagittal sinuses. (E.g., Why is there a significant decrease in signal between baseline and 3h time point in the PSD?) Are there other regions in the image that can be used as a confirmatory reference?
4.From Suppl fig 5, it appears that the signal change peaked in the ventricular CSF and sulcal CSF at different times. Along the lines of point 2 above, it would have been good to see signal changes in the venous sinuses as well as in the cervical lymph nodes along with the PSD and brain parenchyma to fully understand the dynamics of GAD clearance.
5.It appears that there is residual signal in the CSF at 48 hours. Is there a subset of patients that may have been followed beyond the 48 hours reported here? Also, did any patients get any imaging between 6 and 48 hours other than at 24 hours? These time points may help further clarify the dynamics.
6.Please depict the ROI from which signal changes in Fig 1i has been derived. It also appears that some of the ROIs are not in the same location for comparison. It might be better to choose larger ROIs to perform these comparisons.
7.Would it be possible to make a 3D rendering of the arachnoid granulations from the MRI?

Rebuttal letter - Manuscript NCOMMS-19-19161-T

Reviewer #1 (Remarks to the Author, general comment):
Ringstad and Eide apply multi-phase MRI in combination with intrathecal gadobutrol in human subjects and report on a tissue compartment along the superior sagittal sinus a.k.a. "parasagittal dura, PSD" identified on sagittal T2-FLAIR MRIs in 18 subjects.

Major findings and conclusions:
• In human subjects the PSD was identified as a compartment with the following characteristics: 1) intermediate to high signal intensity on T2-FLAIR images after intrathecal gadobutrol; 2) normalized signal intensity significantly higher than adjacent CSF in subarachnoid space on T1-weighted ('black blood', BB) images at 6, 24 and 48 hrs after intrathecal contrast; and 3) the PSD is not intravascular because the BB or blood nulling T1 sequence did not void it out • Signal changes in the PSD was found to be highly correlated with signal in adjacent subarachnoid space at each timepoint after intrathecal contrast delivery • Signal changes in one identified Pacchionian granulation protruding into the dural sinus was also captured with what appears to be a similar time course (peak at 24 hrs).
• Authentic dural lymphatics could not be identified • The authors that 'we demonstrate trans-arachnoid tracer escape from CSF in man". They further speculate that "this observation suggest that molecules drain directly from CSF to parasagittal dura and not primarily along veins exiting the brain"

Response to Reviewer #1, General Comment:
We sincerely thank the reviewer for critically assessing our manuscript, and this is a good summary of our main observations.

Reviewer #1, Comment 1:
The identification of the PSD based on multiphase MRI and intrathecal contrast is indeed novel and intriguing especially since this is an observation in live human brain. The question remains however, what this compartment really represents? i.e. does it truly 'belong' to dura? and/or does it represent a solute draining route as claimed by the authors? Could it be a collection of microscopic arachnoid villi (which cannot be captured at the spatial resolution)? or is it an artifact due to incomplete blood suppression? Answers to these questions are clearly required for further interpretation.

Response to Reviewer #1, Comment 1:
We appreciate these thoughtful comments and are encouraged by the reviewer`s interest in our findings. Indeed, we agree the manuscript would benefit from a closer elaboration of what the PSD represents. In the Brief Communication format, we had to restrain ourselves with regards to number of words, but realize this particular topic should anyhow be discussed more profoundly. The PSD has previously been described in anatomical studies (e.g. in *Fox R et al. Anatomic details of intradural channels in the parasagittal dura: A possible pathway for flow of cerebrospinal fluid (Neurosurgery, Vol. 39, No.1, July 1996)* and *Han H et al. The dural entrance of cerebral bridging veins into the superior sagittal sinus: an anatomical comparison between cadavers and digital subtraction angiograph (Neuroradiology, Vol. 49, Issue 2, Febr 2007)).* Along with this rebuttal letter, we enclose a ppt-file with one slide containing two figure elements retrieved from each of these prior publications. One of these could also serve well for illustration purposes in our manuscript under permission of the publisher. Particularly, Figure 9 in Fox et al. (*Neurosurgery, 1996*) demonstrates well that the PSD "compartment" truly belongs to dura, having a dense carpet of arachnoid granulations medially and being in close association with an extensive network of intradural channels, coursing and coalescing from laterally towards the superior sagittal sinus. The authors hypothesize this may serve as a possible pathway for CSF flow, but has never since been proved due to the lack of a human in vivo tracer study, as we now have carried out. It is true that our image resolution of 1 mm is too large to separate the carpet of arachnoid granulations from the small adjacent veins and their interspersed intradural channels. However, our main point is to show that this is indeed a drainage route in humans, not only for CSF, but also for a hydrophilic molecule such

as our tracer gadobutrol, which shares many of the same traits as neurotoxic solutes such as tau and amyloid-B. Moreover, the area with tracer enhancement stretches more laterally and to a larger extent than what can be anticipated from the anatomical drawings and photographs we have enclosed, which should be highly suggestive for tracer escape to dura even outside arachnoid granulations. To this end, we note that any escape to arachnoid granulations whatsoever has lately been questioned (e.g. as in *Ma Q et al. Outflow of cerebrospinal fluid is predominantly through lymphatic vessels and is reduced in aged mice (Nat Comm, 2017)*, where also the commonly accepted assumption of the impermeable arachnoid is underlined. Our observations in humans are directly contradictive to this animal tracer study. Even though we also observe tracer egress along nervous cranial nerve outlets at the skull base, this was rarely seen towards the nasal lymphatic through the cribriform plate, and was also visually less prominent along other nerve outlets than to the PSD. We try to underline this observation in our Suppl Figure 5.

Furthermore, we acknowledge the reviewer`s concern that the tracer enhancement might represent an artifact due to incomplete blood suppression at T1-BB. However, we must say; we have never experienced this occurring in any blood vessels using the current T1-BB sequence, particularly not with concern to cortical veins, which would definitely have been a problem. Fortunately, we have also acquired T1 weighted GRE images at the same time intervals as T1-BB, demonstrating the same phenomenon (as shown in slide 1). T1-BB images were chosen for ROI measurements as they in most cases are far better for delineation of PSD in contrast to the subarachnoid space. We enclose one example of tracer enhancement at T1 GRE (ppt-file slide 1), which might suit well as basis for a supplementary figure, and we hope this should eliminate the concern of incomplete blood suppression at T1-BB. We are, however, reluctant to base all our ROI measurements on T1 GRE images, as this will reduce our ability to position the ROIs correctly in representative areas.

Reviewer #1, Comment 2:

To this reviewer the most important question in regards to the PSD compartment is whether or not it truly represents a draining route i.e. there is no concrete evidence of this in the presented data.

Response to Reviewer #1, Comment 2:

With reference to our response to the previous comment, we hope the reviewer can now agree with us that evidence of CSF tracer egress to PSD has been provided.

Reviewer #1, Comment 3:

The time course of signal changes captured in the subarachnoid CSF, PSD and Pacchionian granulation are concurrent – it would be important to have the normalized signal changes in the pacchioninan granulation for comparison.

Response to Reviewer #1, Comment 3:

We thank the reviewer for this important comment. In Supplementary Figure 2, we provided only absolute signal units for illustration purposes, but fully agree we should include normalized signal changes, which would be included in a revised version, where a graphic illustration of the signal change along with its counterpart in PSD could be useful.

Reviewer #1, Comment 4:

The T1 weighted BB sequence can be challenging and signal suppression within the vessel lumen is largely dependent on fast arterial blood flow. Because of this dependency, the images are highly susceptible to flow-related artifacts – could the PSD changes be influenced by incomplete blood suppression?

Response to Reviewer #1, Comment 4:

We understand the reviewer`s concern, and if signal suppression at our T1-BB sequence had been unstable or unpredictable, this would of course represent a major flaw. This has not been the case, underlined by our T1 GRE images demonstrating the same effect as in T1-BB. Should there have been

even slow flow in the area, tracer would have been quickly washed out rather than accumulating with a peak occurring typically at 24 hours.

<u>Reviewer #1, Comment 5:</u>

More examples from other subjects of PSD anatomy would be important and informative. Any further volumetric quantification of the PSD compartment in relation to dura is lacking.

Response to Reviewer #1, Comment 5:

With a cohort of 18 human subjects, we have no problem with providing more examples of PSD anatomy, which indeed show some inter-individual variations. Should the Editor allow us to include more display items, or that we extend our manuscript to a full Research Article, we may of course do that, and also address inter-individual PSD volume aspects. As part of our preliminary work on the data, we already looked at what we named “PSD prominence” (grade 1-3), but this didn`t yield information we found worth including in this Brief Communication.

<u>Reviewer #2 (Remarks to the Author, General Comment):</u>

This manuscript is a follow up on the Oslo team explorations of CSF transport in the human brain and surrounding structure. The authors collect repeated scans over 48 hrs after intrathecal delivery of a contrast agent in 18 patients. They report that the contrast agent accumulates in the parasagittal dura. This conclusion is based on clever use of T2-FLAIR, which suppress the signal from the free fluid filled compartments such as the subarachnoid space, whereas T1-BB images darken the contents of blood vessels. The authors define tissue with an intermediate T2-FLAIR signal that are not blood vessels as dura. This tissue with these imaging characteristics was detected almost exclusively along with the middle third of the superior sagittal sinus. The imaging does very convincingly show that the contrast agent accumulates in this space. My major concern is that the evidence for this is a compartment of dura is indirect. Why not image a cadaver and then follow the imaging up with traditional histology? Albeit I am not an expert, the compartment with retention of the contrast agent is huge compared with the sinus and does not look like the classical cross section of dura in medical textbooks. The compartment might be a specialization of the subarachnoid space containing multiple membranes that subdivide the space and reduce the T2-FLAIR. Also, it seems odd that dura which is separated from the subarachnoid space by a barrier of tight junctions would take up and retain so large quantities of the contrast agent.

Response to Reviewer #2, General Comment:

We thank the reviewer for critically revising our manuscript and agree that the contrast agent accumulation in PSD is indeed convincing. The evidence in favor that this represents a compartment of the dura has been provided in previous anatomical studies that previously did not received much attention (probably as no one has later proven CSF- and tracer egress towards it) , and we have enclosed two of these previous papers along with this rebuttal letter. For a further discussion, we refer to our previous answer to **Reviewer #1, Comment 1.** We hope this should alleviate the need of a cadaver study, which is also beyond reach of our clinical research group. We hope, however, that our novel, and quite consistent findings made in living humans may spark a new interest for this area in subsequent cadaver and animal studies.

The most understandable argument that the dura should be separated from the subarachnoid space by a barrier of tight junctions, really underlines the importance of our observations, which directly contradict the impermeability of the arachnoid membrane, at least in this particular region.

<u>Reviewer #3 (Remarks to the Author, General Comment):</u>

In the manuscript “Cerebrospinal fluid tracer efflux to parasagittal dura in humans” the authors describe experiments to demonstrate the clearance of CSF from the brain using MRI contrast agent

(Gad) introduced into the CSF compartment in humans. Follow-up for 2 days show enhancement of the parasinal dura (PSD), which peaked at about one day after injection of the Gad. Experiments such as the one described here is critical in better understanding of CSF clearance and lymphatic access, which may have implications in many diseases. However, a few clarifications are required before the article can be accepted:

Response to Reviewer #3, General Comment:

We sincerely thank the reviewer for these encouraging comments regarding the importance of our work and agree this is an important step forward in better understanding of CSF clearance routes and the link to lymphatic vessels in the dural sinus wall. We are happy to provide the needed clarifications as follows:

Reviewer #3, Comment 1:

The CSF is not black in the pre-Gad T1 black-blood (BB) images (Fig 1c, suppl fig 1a, suppl fig 2a), indicating that these might be more like a PD image. Please clarify.

Response to Reviewer #3, Comment 1:

In the T1-BB sequences, signal is nulled in areas with moving protons, such as in arteries and veins. Around the high brain convexities, there are little motions within the CSF, and therefore the signal is not black (nulled) as in the blood vessels, rendering for a more intermediate CSF-signal. Indeed, there is some loss of signal in the basal CSF cisterns around the brain stem (Suppl Figure 5) due to faster, pulsatile CSF flow, but this does not affect CSF spaces under the cranial vertex.

Reviewer #3, Comment 2:

The pulse sequences used in the manuscript is limited to FLAIR and T1BB. It would have been immensely useful to have a T1-map or even a GRE T1-weighted image to quantify these changes. It might have been possible to (at least partially) quantify the GAD in the sagittal sinuses using GRE sequence.

Response to Reviewer #3, Comment 2:

We thank the reviewer for this important comment that has made us realize the study would benefit from being substantiated with T1-weighted images, in addition to T1-BB. Fortunately, we had already obtained T1 GRE images at the same time intervals as T1-BB. We use T1 GRE for analysis of tracer enhancement in brain tissue, from which our group has published several previous studies. The T1 GRE images are, however, not as ideal as T1-BB in delineating the subarachnoid space from the PSD, but we enclose with this rebuttal letter a ppt-presentation (slide 1) where the same phenomenon is shown in both T1-BB and T1 GRE images. This could serve well as a basis for a supplementary figure if the Editor allows us to extend the number of display figures. The amount of gadobutrol in CSF is far too low to show any enhancement effect within the systemic blood circulation at level of the sagittal sinus (as previously shown by *Ringstad et al. Glymphatic MRI in Idiopathic Normal Pressure Hydrocephalus (Brain, 2017)*).

Reviewer #3, Comment 3:

Along these lines, normalization with vitreous signal in the eye was a good idea for lineally changing signal. However, this may not be the case here as scans were performed in multiple sessions, especially with dramatic contrast changes in the brain. It is conceivable that there are non-linear effects from B1 and B0 shimming, that affect the ocular region differently than the region of the sagittal sinuses. (E.g., Why is there a significant decrease in signal between baseline and 3h time point in the PSD?) Are there other regions in the image that can be used as a confirmatory reference?

Response to Reviewer #3, Comment 3:

This insightful comment touches upon a challenge we have had to address in several previous studies: MR images are susceptible to shifts in baseline signal unit level, and in our experience, this mostly may occur when contrast enrichment in CSF is most prominent, typically in the early arrival phase in the intracranial compartment. Normalization of signal units is therefore needed to

compensate for this. However, as the contrast agent (CSF tracer) in the end is "everywhere" intracranially, there are few areas left amenable as reference tissue. We have chosen the ocular bulb, as the size of this region is robust and homogenous with signal intensity level close to the areas of interest (CSF and PSD). We have tested other regions as well, such as ocular muscles and within the superior sagittal sinus, but which do not appear to be as stable as the bulbs. It is therefore highly unlikely that other reference regions would improve the precision level nor in any way change the conclusions of this study.

<u>Reviewer #3, Comment 4:</u>

From Suppl fig 5, it appears that the signal change peaked in the ventricular CSF and sulcal CSF at different times. Along the lines of point 2 above, it would have been good to see signal changes in the venous sinuses as well as in the cervical lymph nodes along with the PSD and brain parenchyma to fully understand the dynamics of GAD clearance.

Response to Reviewer #3, Comment 4:

As already commented, previous studies have shown no sign of enhancement in the venous sinuses. We have also published a study showing CSF tracer enhancement in neck lymph nodes with peak typically after 24 hours (*Eide et al. Magnetic Resonance Imaging Provides Evidence of Glymphatic Drainage from Human Brain to Cervical Lymph Nodes (Scientific Reports, 2018)*). In the present study, MRI of the neck was not included. The brain parenchyma is a whole topic at its own, and we have published an extensive data set showing tracer uptake in all brain regions typically peaking at 24 hours (*Ringstad et al. Brain-wide Glymphatic Enhancement and Clearance in Humans Assessed with MRI (JCI Insight, 2018)*). A further assessment of brain tissue enhancement here is not expected to bring anything new to the field and would by far exceed the extent of the Brief Communication format in Nat Comm. We hope it is therefore acceptable that we refer to our previous studies for a closer overview of GAD clearance dynamics.

<u>Reviewer #3, Comment 5:</u>

It appears that there is residual signal in the CSF at 48 hours. Is there a subset of patients that may have been followed beyond the 48 hours reported here? Also, did any patients get any imaging between 6 and 48 hours other than at 24 hours? These time points may help further clarify the dynamics.

Response to Reviewer #3, Comment 5:

We fully acknowledge the reviewer`s desire for more temporal information. Unfortunately, we do not have imaging data from time points later than 48 hours or between 6 and 24 hours, which can already be regarded as very prolonged imaging, and quite unique. The extensive imaging routine already put a major strain on our study patients, and this has limited us from more extensive imaging in this round.

<u>Reviewer #3, Comment 6:</u>

Please depict the ROI from which signal changes in Fig 1i has been derived. It also appears that some of the ROIs are not in the same location for comparison. It might be better to choose larger ROIs to perform these comparisons.

Response to Reviewer #3, Comment 6:

Figure 1 was meant purely for illustration purposes of the concept. The ROIs are demonstrated in Supplementary Figures 1 and 2, respectively. We are of course willing to include ROIs also in Figure 1 if this is still considered needed. The size of the ROIs represents a compromise where we aimed to include as many pixels as possible and still avoid partial volume effects towards the periphery. As the signal increase in PSD can be considered homogenous, we would rather keep partial volume effects to a minimum instead of increasing ROI size.

<u>Reviewer #3, Comment 7:</u>

Would it be possible to make a 3D rendering of the arachnoid granulations from the MRI?

Response to Reviewer #3, Comment 7:

This can of course be done, our T1-BB images are volume acquisitions and well suited for this purpose, probably best at the 24 hour time point, where image contrast between the AG and sagittal sinus is high.

REVIEWERS' COMMENTS:

Reviewer #1 (Remarks to the Author):

The resubmitted and revised manuscript by Ringstad and Eide “Cerebrospinal fluid tracer efflux to parasagittal dura in humans” has greatly improved with the added data/figures and text.
Most of this reviewer’s comments and requests for improvements have been addressed and the findings are indeed novel especially because it is an in vivo finding in humans. It sheds new light on CSF transport dynamics in the live human brain.

There are still two issues I would like to see addressed or implemented:

1. Ringstad and Eide are both experts in the field of CSF flow dynamics in the human brain and would acknowledge (at least this reviewer would think so) that CSF is constantly produced and reabsorbed. However, it is unknown how much of CSF is transported from (the site of production mostly choroid plexus) or from the basal cisterns is passing through brain parenchyma before it exits the calvarium. In other words CSF can ‘bypass’ the parenchyma having never exchanged with the brain interstitial fluid space. Therefore, the authors are correct when being succinct with respect to their stated the title “CSF tracer efflux to parasagittal dura in humans”. On the other hand, the section on ‘possible link between glymphatic circulation and dural lymphatic vessels, where it is stated …”strongly suggest that molecules drain directly from CSF, and not primarily along veins exiting the brain” is an over-interpretation (and likely erroneous) because their data stream cannot address this critical issue as there is no parenchymal information given. In other words, it is possible that the CSF solute entering the PSD lacunaes in the dura have never exchanged with interstitial fluid and therefore is not representative of glymphatic efflux.

2. Regarding the statement pertaining to the transnasal lymphatic route i.e. “the presumed importance of the transnasal lymphatic route seems less in humans, as signs of tracer enhancement below the cribriform plate could be visually confirmed in only 2/18 patients” I would also respectfully caution the authors on the validity of this sentence given the poor time resolution of the of the study, and given that all the tracer tracked may not represent efflux from the glymphatic pathway per se. In other words, the inability to detect tracer efflux below the cribriform plate may be related to signal-to-noise and/or lack of temporal resolution. There are amble studies in the literature showing that the transnasal lymphatic route may be important.

Reviewer #2 (Remarks to the Author):

The resubmitted article by Ringstad and Eide is easier to follow than the original article. In part because it connects better to the prior published publication by Fox et al. The imaging data provided by Ringstad and Eide is novel in that they provide functional data demonstrating that a compartment - the parasagittal dura – exists and that this compartment is separate from the subarachnoid space/CSF. However, the study does not show that CSF efflux occurs via this compartment. This fact needs to be stated clearly in the abstract. In future work, it would be important to explore how the parasagittal dura changes in hydrocephalus and other pathologies such as in subarachnoid bleeding. The novel findings may also be important for understanding the pathophysiology of migraine. In sum, the authors have addressed all my concerns and their study provides a significant advance as it documents the existence of a compartment of the human CNS that has not been described functionally before.

Reviewer #3 (Remarks to the Author):

The authors have responded to my questions in a satisfactory manner.

Comments to the report of Reviewer #1 (NCOMMS-19-19161B)

Reference is to manuscript with tracked changes

Remarks to the Author, general comment:
The resubmitted and revised manuscript by Ringstad and Eide "Cerebrospinal fluid tracer efflux to parasagittal dura in humans" has greatly improved with the added data/figures and text.
Most of this reviewer's comments and requests for improvements have been addressed and the findings are indeed novel especially because it is an in vivo finding in humans. It sheds new light on CSF transport dynamics in the live human brain.
There are still two issues I would like to see addressed or implemented:
Response to Reviewer #1, General Comment:
We thank the reviewer for this positive comment. The two issues are addressed below.

Comment #1:
1. Ringstad and Eide are both experts in the field of CSF flow dynamics in the human brain and would acknowledge (at least this reviewer would think so) that CSF is constantly produced and reabsorbed. However, it is unknown how much of CSF is transported from (the site of production mostly choroid plexus) or from the basal cisterns is passing through brain parenchyma before it exits the calvarium. In other words CSF can 'bypass' the parenchyma having never exchanged with the brain interstitial fluid space. Therefore, the authors are correct when being succinct with respect to their stated the title "CSF tracer efflux to parasagittal dura in humans". On the other hand, the section on 'possible link between glymphatic circulation and dural lymphatic vessels, where it is stated ..."strongly suggest that molecules drain directly from CSF, and not primarily along veins exiting the brain" is an over-interpretation (and likely erroneous) because their data stream cannot address this critical issue as there is no parenchymal information given. In other words, it is possible that the CSF solute entering the PSD lacunaes in the dura have never exchanged with interstitial fluid and therefore is not representative of glymphatic efflux.
Response to Comment #1:
This is a relevant discussion raised by the Reviewer. We base our reasoning on a strong association between CSF tracer enhancement within CSF and nearby parasagittal dura (Fig. 4). In previous studies, we have shown a strong association between CSF tracer enhancement in CSF and nearby parenchyma (see References 1 and 23). Furthermore, it has been shown in rats that maximum parenchymal tracer uptake was 19 % of the total amount delivered to cisterna magna (Lee H et al. Quantitative Gd-DOTA uptake from cerebrospinal fluid into rat brain using 3D VFA-SPGR at 9.4T (Magnetic Resonance in Medicine 79:1568-1578 (2018)). Indeed, there must be differences dependent on species, injection site and tracer dose, but extra-vascular efflux of molecules directly from CSF to lymphatic pathways still seems to have a major role. We thank the reviewer for bringing up this important aspect and have added the reference (new ref 24), and modified the sentence to be more cautious about this aspect (page 9, para 2).

Comment #2:
2. Regarding the statement pertaining to the transnasal lymphatic route i.e. "the presumed importance of the transnasal lymphatic route seems less in humans, as signs of tracer enhancement below the cribriform plate could be visually confirmed in only 2/18 patients" I would also respectfully caution the authors on the validity of this sentence given the poor

time resolution of the of the study, and given that all the tracer tracked may not represent efflux from the glymphatic pathway per se. In other words, the inability to detect tracer efflux below the cribriform plate may be related to signal-to-noise and/or lack of temporal resolution. There are amble studies in the literature showing that the transnasal lymphatic route may be important.

Response to Comment #2:

We thank the Reviewer for this comment, and agree that the observations of this work do not support this statement. The sentence has been modified accordingly (page 8, last para).

Comments to the report of Reviewer #2 (NCOMMS-19-19161B)

Remarks to the Author, General Comment:

The resubmitted article by Ringstad and Eide is easier to follow than the original article. In part because it connects better to the prior published publication by Fox et al. The imaging data provided by Ringstad and Eide is novel in that they provide functional data demonstrating that a compartment - the parasagittal dura – exists and that this compartment is separate from the subarachnoid space/CSF. However, the study does not show that CSF efflux occurs via this compartment. This fact needs to be stated clearly in the abstract. In future work, it would be important to explore how the parasagittal dura changes in hydrocephalus and other pathologies such as in subarachnoid bleeding. The novel findings may also be important for understanding the pathophysiology of migraine. In sum, the authors have addressed all my concerns and their study provides a significant advance as it documents the existence of a compartment of the human CNS that has not been described functionally before.

Response to General Comment:

We thank Reviewer #2 for thorough review of our work. Regarding the comment to state in the abstract that passage of CSF to parasagittal dura is not shown in this study, we follow the instructions of the Editor. We fully agree on the possible implications addressed by the Reviewer, and this is something we will endeavor to explore further in future work.

Comments to the report of Reviewer #3 (NCOMMS-19-19161B)

Remarks to the Author, General Comment:

The authors have responded to my questions in a satisfactory manner.

Response to General Comment:

We thank Reviewer #3 for thorough review of our work.
